# Regulation of autoimmune arthritis by the SHP-1 tyrosine phosphatase

**DOI:** 10.1186/s13075-020-02250-8

**Published:** 2020-06-26

**Authors:** Adrienn Markovics, Daniel M. Toth, Tibor T. Glant, Katalin Mikecz

**Affiliations:** Department of Orthopedic Surgery, Section of Molecular Medicine, Rush University Medical Center, 1735 W. Harrison Street, Cohn Research Building, Room 741, Chicago, IL 60612 USA

**Keywords:** Rheumatoid arthritis, Autoimmunity, Tyrosine phosphatase, Proteoglycan-induced arthritis, T cells

## Abstract

**Background:**

The Src homology region 2 domain-containing phosphatase-1 (SHP-1) is known to exert negative regulatory effects on immune cell signaling. Mice with mutations in the *Shp1* gene develop inflammatory skin disease and autoimmunity, but no arthritis.

We sought to explore the role of SHP-1 in arthritis using an autoimmune mouse model of rheumatoid arthritis. We generated *Shp1* transgenic (*Shp1*-Tg) mice to study the impact of SHP-1 overexpression on arthritis susceptibility and adaptive immune responses.

**Methods:**

SHP-1 gene and protein expression as well as tyrosine phosphatase activity were evaluated in spleen cells of transgenic and wild type (WT) mice. WT and *Shp1*-Tg (homozygous or heterozygous for the transgene) mice were immunized with human cartilage proteoglycan (PG) in adjuvant, and arthritis symptoms were monitored. Protein tyrosine phosphorylation level, net cytokine secretion, and serum anti-human PG antibody titers were measured in immune cells from WT and *Shp1*-Tg mice. WT mice were treated with regorafenib orally to activate SHP-1 either before PG-induced arthritis (PGIA) symptoms developed (preventive treatment) or starting at an early stage of disease (therapeutic treatment). Data were statistically analyzed and graphs created using GraphPad Prism 8.0.2 software.

**Results:**

SHP-1 expression and tyrosine phosphatase activity were elevated in both transgenic lines compared to WT mice. While all WT mice developed arthritis after immunization, none of the homozygous *Shp1*-Tg mice developed the disease. Heterozygous transgenic mice, which showed intermediate PGIA incidence, were selected for further investigation. We observed differences in interleukin-4 and interleukin-10 production in vitro, but serum anti-PG antibody levels were not different between the genotypes. We also found decreased tyrosine phosphorylation of several proteins of the JAK/STAT pathway in T cells from PG-immunized *Shp1*-Tg mice. Regorafenib administration to WT mice prevented the development of severe PGIA or reduced disease severity when started after disease onset.

**Conclusions:**

Resistance to arthritis in the presence of SHP-1 overexpression likely results from the impairment of tyrosine phosphorylation (deactivation) of key immune cell signaling proteins in the JAK/STAT pathway, due to the overwhelming tyrosine phosphatase activity of the enzyme in *Shp1*-Tg mice. Our study is the first to investigate the role of SHP-1 in autoimmune arthritis using animals overexpressing this phosphatase. Pharmacological activation of SHP-1 might be considered as a new approach to the treatment of autoimmune arthritis.

## Background

The Src homology region 2 domain-containing phosphatase 1 (SHP-1, encoded by the *Shp1/Ptpn6* gene) is a non-receptor tyrosine phosphatase expressed in hematopoietic cells and, at a lower level, in epithelial cells. It is known to exert a negative regulatory effect on cellular signaling in cells of both the innate and adaptive immune systems [1]. SHP-1 has been shown to be recruited to receptors with immunoreceptor tyrosine-based inhibition motifs (ITIMs) [2] and to act on receptors bearing immunoreceptor tyrosine-based activation motifs (ITAMs) as well as on intracellular protein kinases involved in the initiation of signaling cascades in T and B cells upon activation of the T cell and B cell receptors (TCR, BCR), respectively [3–6]. SHP-1 can dephosphorylate (thus potentially deactivate) a number of kinases including Lck-56, Zap-70, Lyn, and Syk [7–12]. SHP-1 has 2 tandem SH2 domains that allow the enzyme to bind to ITIMs, and its catalytic protein tyrosine phosphatase domain then dephosphorylates specific tyrosine residues within the SHP-1-bound substrate [13]. An earlier study has determined that SHP-1 is subject to autoregulation by folding its SH2 domains upon the catalytic domain, as SHP-1 isoforms lacking the N-terminal SH2 domain or having a mutation in the C-terminal SH2 domain had much higher phosphatase activity than the intact enzyme [14]. A more recent study has shown that regorafenib, an anti-cancer drug, was able to inhibit the tyrosine phosphorylation-related activity of oncogenic transcription factors via increased dephosphorylation by relieving the autoinhibitory conformation, i.e., by increasing the phosphatase activity of SHP-1 [15].

Much of the information about the role of SHP-1 in the regulation of the activity of immune cells has been provided by studies on mice with spontaneously arising mutations in the *Shp1* gene [16]. The absence or diminished catalytic function of SHP-1 manifests in the motheaten (me) phenotype in mice, and its genetic polymorphism plays a role in neutrophilic dermatoses in humans [17]. Recent experiments with a conditional deletion of *shp1* in neutrophils showed that SHP-1 had a dual function in these innate immune cells, as it inhibited p38 MAPK function and consequently limited the production of pro-inflammatory cytokines such as TNF and IL-1 [18]. The Ptpn6^meB2/meB2^ (meB2) condition develops due to the spontaneous insertion of a B2 element in exon 6 of the *Shp1* gene [19]. Mice with the *Shp1* mutation are characterized by patchy absence of hair (hence the motheaten phenotype) and massive skin inflammation as well as hypergammaglobulinemia and systemic autoimmunity [16, 19], but the joints are not affected with arthritis. In support of the negative regulatory role of SHP-1 in immune cell signaling, lymphocytes in me mice have been shown to be hyper-reactive to either TCR or BCR stimulation [4, 5]. Additionally, T cell-specific deletion of the *Shp1* gene promoted T helper 17 (Th17) cell differentiation [20], while B cell-specific ablation of this gene led to the production of autoantibodies (autoAbs) and autoimmune glomerulonephritis in mice [21]. In myeloid cells, SHP-1 regulates many different signaling pathways [22]. Despite the involvement of SHP-1 in the signaling pathways of various immune cells, its role has not been investigated in murine models of rheumatoid arthritis (RA).

Cartilage proteoglycan (PG)-induced arthritis (PGIA) is a well-characterized animal model of RA that shares many pathological and autoimmune features as well as genetic risk loci with the human disease [23–27]. The autoimmune aspects of PGIA include T cell and B cell (serum Ab) reactivity with self (mouse) PG [25] as well as with arginine- (R49) and citrulline (C49)-containing forms of a PG autoepitope (whose sequences are identical in humans and mice) [28].

Our goals were to examine how SHP-1 affects adaptive immune responses in *Shp1* overexpressing mice and autoimmune arthritis using the PGIA model. We also investigated the effects of pharmacological activation of the enzyme on arthritis in mice with PGIA.

## Methods

### Generation of *Shp1*-transgenic mice

The *Shp1* gene is located in a high-density locus on mouse chromosome 6. In order to investigate the effect of *Shp1* overexpression, we generated *Shp1*-transgenic (Tg) mice. Briefly, we purchased BAC clone RP24-297C1 containing the full-length *Shp1* gene with its promoter region from BACPAC Resources Center (Children’s Hospital Oakland Research Institute in Oakland, CA). The 21.9 Kbp genomic fragment containing *Shp1* (derived from a C57Bl/6 mouse) was released from the BAC clone by digestion with *Fspl* restriction enzyme and separated from other DNA fragments in 0.5% agarose gel by pulse-field electrophoresis (Bio-Rad, Hercules, CA). The 21.9 Kbp fragment was further purified using GeneClean Spin kit (BIO101 Systems, Carlsbad, CA) and sequenced. The purified fragment, containing the *Shp1* gene, its promoter, and 2 Kbp flanking regions, was sent to the Cornell University Core Transgenic Mouse Facility (Ithaca, NY) for pronuclear injection into fertilized FVB mouse egg cells. We identified a simple sequence length polymorphism (SSLP) in the 5′ flanking region of the *Shp1* transgene (from the donor C57Bl/6 mouse), which was not present in FVB (origin of the fertilized egg) or BALB/c cells. This SSLP was located at a *Taqα*I restriction enzyme-sensitive site, which allowed us to use appropriate primers that could discriminate between the *Shp1* transgene (*Taqα*I-resistant) and the endogenous *Shp1* gene (*Taqα*I-sensitive) in FVB or BALB/c mice. We identified tandem inserts of the *Shp1* transgene with its promoter in mouse chromosome 7.

Transgenic founders were backcrossed into the BALB/c (PGIA-susceptible) background for 12 generations. Heterozygous *Shp1*-Tg BALB/c mice were then intercrossed to generate *Shp1*-Tg^+/−^, *Shp1*-Tg^+/+^ and WT offspring.

### Confirmation of *Shp1* gene expression

*Shp1* gene expression was quantified by RT-qPCR from spleen cells of WT as well as heterozygous (*Shp1*-Tg^+/−^) and homozygous (*Shp1*-Tg^+/+^) Tg mice. Female BALB/c mice at 8–12 weeks of age of each genotype were used. Total RNA was extracted with Direct-Zol RNA MiniPrep Plus (Zymo Research, Irvine, CA) and reverse transcribed using the iScript Reverse Transcription Supermix for RT-qPCR (Bio-Rad, Hercules, CA). cDNA was amplified using SsoAdvanced Universal SYBR Green Supermix (Bio-Rad) in a CFX Connect Real-Time PCR Detection System (Bio-Rad). Measured C_t_ values were normalized to the housekeeping hypoxanthine phosphoribosyltransferase 1 (*Hprt1)* gene. Relative expression was calculated using CFX Manager Software (Bio-Rad). *Shp1* primer sequences were as follows:

Forward: 5′-TCT CAG TCA GGG TGG ATG AT-3′

Reverse: 5′-CCT GCT GCT GCG TGT AAT A-3′

### Detection of SHP-1 protein expression by Western blot

Cellular proteins were extracted and the protein concentrations were determined using a BCA Protein Assay kit (ThermoFisher, Rockford, IL) and a Synergy 2 ELISA reader (BioTek Instruments, Winooski, VT). Protein extracts were separated on 4–20% SDS-polyacrylamide gels (Bio-Rad), blotted onto PVDF membranes (Bio-Rad), then blocked with Tris-buffered saline + 0.05% Tween-20 containing 3% blotting-grade blocker (Bio-Rad), and incubated with the following primary antibodies at 4 °C overnight: anti-SHP-1, (GTX20658 GeneTex, Irvine, CA), and anti-GAPDH (G9545 Sigma-Aldrich, St Louis, MO). Horseradish peroxidase (HRP)-conjugated secondary antibodies were purchased from Santa Cruz Biotechnology (Dallas, TX). Pierce ECL Western Blotting Substrate (ThermoFisher, Rockford, IL) was used to generate chemiluminescence signals, which were detected on X-ray films.

### Tyrosine phosphatase activity assay

SHP-1 was immunoprecipitated from whole cell lysates using anti-mouse SHP-1 antibody (GeneTex) and Protein A/G Plus-Agarose beads (Santa Cruz Biotechnology) following the manufacturer’s protocol. SHP-1 tyrosine phosphatase activity was measured with RediPlate 96 EnzCheck Tyrosine Phosphatase Assay Kit (Molecular Probes, Eugene, OR) according to the manufacturer’s instructions. Fluorescence was measured in an ELISA reader (BioTek Instruments), and tyrosine phosphatase activity was expressed as the percent of phosphatase activity detected in cell lysates of WT mice.

### Flow cytometric analysis of B and T cell populations in the spleen

The spleens were harvested from the mice (*n* = 4 per genotype). Cells were released by pressing the spleens under sterile metal screens and erythrocytes were lysed in cold ammonium chloride-based buffer. Fc receptors were blocked with TruStain FcX anti-mouse CD16/CD32 (cat# 101320; BioLegend, San Diego, CA), and surface antigens were stained with fluorescence-labeled antibodies to mouse CD3, CD4, CD8, B220/CD45, IgM, CD21/35, CD23, CD19, and CD138 (Additional file 2). Data acquisition and analysis were performed as before [28] using a FACSCanto II instrument and BD FACSDiva software (version 5.0).

### Polyclonal activation of T cells with anti-mouse CD3/CD28 antibodies

For polyclonal activation, T cells were cultured in the presence of antibodies against mouse CD3 and CD28 (LEAF purified anti-mouse CD3ε antibody, Cat#100314, LEAF purified anti-mouse CD28 antibody, Cat#102112, both from BioLegend). Briefly, the day before harvesting spleen cells, 96-well culture microplates were coated with sterile 0.15 M sodium carbonate coating buffer (pH 9.6) containing 1 μg anti-CD3 and 1 μg anti-CD28 antibodies per well in 100 μl volume. Control wells contained only carbonate buffer. Plates were incubated overnight at room temperature. On the day of harvesting spleen cells, wells were washed 3 times with sterile phosphate-buffered saline (PBS, pH 7.2) and blocked with Dulbecco’s modified Eagle’s medium (DMEM, Sigma-Aldrich) containing antibiotics/antimycotics and supplemented with 10% fetal bovine serum (FBS, Hyclone, Logan, UT). The spleens from *n* = 3–6 mice were harvested and the spleen cells prepared as described above. “Untouched” T cells were isolated using immunomagnetic negative selection (EasySep Mouse T cell isolation kit, StemCell Technologies, Vancouver, Canada).

For proliferation assays, purified T cells were suspended in DMEM supplemented with 10% FBS and seeded in the culture microplates (pre-coated with anti-mouse CD3 and CD28 or coating buffer only) at a density of 2 × 10^5^ viable cells in 200 μl of medium per well in triplicate wells. The cultures were maintained for 5 days in a culture incubator at 37 °C with 5% CO_2_ in air. For determination of cell proliferation, 18–20 h before harvest, the cells were pulsed with 0.5 μCi/well of [^3^H] thymidine (Perkin Elmer, Boston, MA). Isotope incorporation was measured using a scintillation counter (Micro-Beta; Perkin Elmer) and expressed as counts per minute (cpm). Stimulation index (SI) was calculated by dividing the mean cpm values of stimulated wells with the mean cpm values of control wells.

### Activation of B cells with anti-mouse IgM antibody

To assess the polyclonal activation of B cells in WT and *Shp1*-Tg genotypes, we set up B cell cultures from WT, *Shp1*-Tg^+/+^, and *Shp1*-Tg^+/−^ mice and used anti-mouse IgM for activation (anti-mouse IgM F (ab’)2, μ chain-specific, Functional Grade Purified, eBioscience, cat# 16-5092). Spleen cells of *n* = 3–6 mice per genotype were harvested. B cells were isolated using immunomagnetic negative selection (EasySep mouse pan B cell isolation kit, StemCell Technologies).

For proliferation assays, B cells were suspended in DMEM supplemented with 10% FBS and seeded in 96-well culture microplates at a density of 2 × 10^5^ viable cells in 200 μl of medium per well. Anti-IgM (10 μg/ml) was added to stimulated wells, while control wells contained only medium and the cells (both in triplicates). The culture was maintained for 5 days_._ [^3^H] thymidine (Perkin Elmer) incorporation was measured as described for T cells. Stimulation index (SI) was calculated by dividing the mean cpm values of stimulated wells with the mean cpm values of control wells.

### Mice, immunization, and visual assessment of PGIA

Human cartilage PG protein was prepared as described [23] to induce autoimmune arthritis. Adult WT (retired breeder) female BALB/c mice were obtained from the National Cancer Institute (NCI; Frederick, MD) or from the NCI colony of Charles River (Wilmington, MA). *Shp1*-Tg mice (both homozygous and heterozygous for the transgene) were bred and kept in the animal facility of Rush University Medical Center under specific pathogen-free conditions. All animal protocols were approved by the Institutional Animal Care and Use Committee (Rush University, Chicago, IL, IACUC permit #17-039).

To induce PGIA, mice were immunized intraperitoneally (i.p.) with human PG (100 μg protein in 100 μl sterile PBS emulsified with dimethyl-dioctadecyl ammonium bromide adjuvant (DDA; Sigma-Aldrich) three times, 3 weeks apart as described [29] [30]. In one of the experiments, mice received four PG injections. Mice were inspected for signs of arthritis (swelling and redness) twice a week after the second PG immunization. Upon disease onset, the degree of arthritis for each paw was visually scored every other day on a scale of 0 to 4 for each limb, summing the individual paw scores to a maximum visual arthritis (VA) score (arthritis severity) of 16 per animal [25] [29]. Arthritis incidence was expressed as the percentage of mice showing arthritis symptoms (> 0.5 score on any limb) of all mice examined in each group.

### Measurement of cytokines in cell culture supernatants

In order to determine net cytokine secretion (stimulated minus unstimulated wells) spleen cells of naïve and PG-immunized mice were harvested, suspended in DMEM supplemented with 10% FBS, and dispensed into 48-well culture plates (2 × 10^6^ viable cells in 900 μl medium per well). Cells were cultured in the absence or presence of human PG (25 μg/ml). On day 4 of culture, the supernatants were collected. Soluble IFNγ, IL-17, IL-10, and IL-4 concentrations were measured using murine cytokine ELISA kits (R & D Systems, Minneapolis, MN) according to the manufacturer’s instructions. Briefly, 96-well Maxisorp ELISA plates (Thermo Scientific, Rochester, NY) were coated with the purified anti-cytokine capture antibodies at the recommended concentration overnight. After repeated washing, free binding sites were blocked with a blocking buffer containing 1% bovine serum albumin (BSA; Sigma-Aldrich). Undiluted cell culture supernatants (100 μl/well) and serially diluted cytokine standards were then incubated in duplicate wells with the immobilized capture antibody for 2 h. After repeated washing, HRP-conjugated anti-cytokine detection antibody was applied to the wells. The color reaction was developed with 3,3′,5,5′-tetramethylbenzidine (TMB) substrate (BD Biosciences). The optical density (OD) values at 450 nm and corresponding cytokine concentrations were determined. Net cytokine secretion was expressed as pg/ml in PG-stimulated minus non-stimulated cells’ supernatants.

### Anti-human PG antibody ELISA

For the measurement of serum anti-human PG antibodies, wells of 96-well ELISA plates (Nunc) were coated overnight with human PG (1 μg/well each) in 100 μl/well of sodium carbonate buffer at room temperature [31]. Unbound antigen was removed by washing with 0.05% Tween 20 in PBS. Wells were blocked with 1.5% non-fat milk in 250 μl/well PBS for 1 h at room temperature on a shaker platform. Serum samples were diluted to 1:100 in blocking buffer and incubated with the antigen-coated wells (100 μl/well, duplicate wells) for 2 h at room temperature with shaking. Bound IgG was detected by incubation with 100 μl/well of HRP-conjugated anti-mouse IgG (BD Biosciences) at a 1:2000 dilution for 2 h at room temperature with shaking. Unbound material was removed with wash buffer between each of these steps. The color reaction was developed with 100 μl/well of *O*-phenylene-diamine solution (Sigma-Aldrich) dissolved freshly in PBS with H_2_O_2,_ and incubation for 30 min in the dark. The reaction was stopped with 25 μl/well stop solution (4 N H_2_SO_4_). Absorbance at 450 nm was read in an ELISA reader.

### Phosphorylation-specific protein microarray analysis

To determine which proteins were tyrosine-phosphorylated differentially in the CD4^+^ T cells of WT versus *Shp1*-Tg mice, Tyrosine Phosphorylation ProArray was performed by the Full Moon Biosystems (Sunnyvale, CA). This array uses site-specific phospho-tyrosine antibodies allowing determination of tyrosine phosphorylation of proteins at 228 specific sites. The array contained 6 technical replicates for each phosphorylation site. Spleen cells were harvested from PG-immunized WT and *Shp1*-Tg^+/−^ mice in 3 independent experiments. Cells were pooled from 5 mice per genotype (a total of 15 WT and 15 Shp1-Tg^+/−^ mice were used). Spleen cells were separated using EasySep magnetic separation kits to obtain CD4^+^/CD25^−^ cell populations. Cell pellets were snap frozen and sent on dry ice to the Full Moon Biosystems for analysis. For data analysis, the intensity of protein phosphorylation in the array was measured using GenePix Pro 6.0 software (Axon Instruments, Molecular Devices, San Jose, CA). The normalized data for each array were computed as follows: normalized data = (Average Signal Intensity of Replicate Spots)/(Median Signal of the Average Signal Intensity) for all antibodies on the array. The normalized data were then used to determine the differences (fold change) between the control (WT) and *Shp1*-Tg samples. Proteins of CD4^+^ cells showing the highest degree of decrease in Tyr phosphorylation were selected. Statistical overrepresentation test was performed using the Panther Classification System v14.0 (http://www.pantherdb.org/) to annotate signaling pathways to the most underphosphorylated proteins. The reference set contained the genes of proteins included in the microarray.

### In vivo SHP-1 activation

In order to investigate if the SHP-1 activator regorafenib (Selleckchem, Houston, TX) has an effect on PGIA development, we administered it preventively to WT BALB/c mice via oral gavage, daily at 10 and 20 mg/kg doses starting at the day of the 3rd PG injection. Regorafenib doses were chosen based on literature provided by the manufacturer, reporting beneficial effects on tumor growth in mice [32]. Vehicle (2% DMSO, 30% PEG300, and 5% Tween 80 in water) served as control. Drug administration and visual arthritis scoring was continued for 24 days after the last immunization. PGIA incidence and severity, in vitro cytokine secretion, and the presence of anti-human PG antibodies (in serum) were evaluated. *Shp1*-Tg^+/−^ mice immunized with PG but without activator treatment were included for reference. In a second setting, 15 mg/kg regorafenib was administered therapeutically, 5 times a week via oral gavage after the initial signs of arthritis developed. Treatment was continued until day 27, and arthritis severity and body weight were monitored.

### Statistical analysis

Data were analyzed and graphs created using Prism 8.0.2 software (GraphPad, USA). Results are expressed as the mean ± SEM unless specified otherwise. Groups were compared using one-way analysis of variance (ANOVA) or Student’s *t* test, and groups affected by two factors were compared by two-way ANOVA followed by Dunn’s or Tukey’s multiple comparison test as appropriate. For time-course experiments, area under the curve (AUC) calculations were also used. In each case, *p* < 0.05 was set as threshold for statistical significance.

## Results

### Expression and tyrosine phosphatase activity of SHP-1 in WT and *Shp1*-Tg mice

**Fig. 1:**
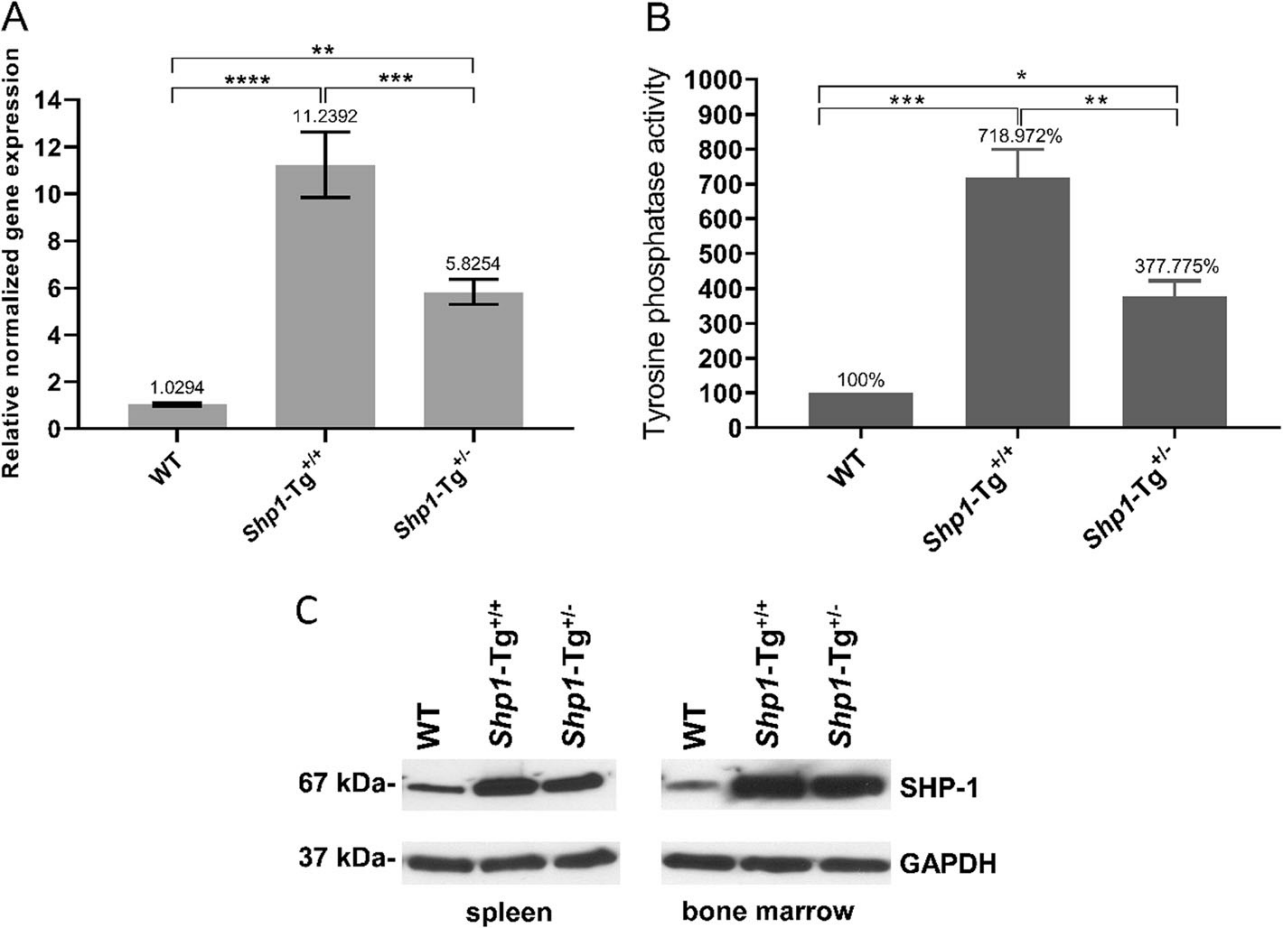
Gene and protein expression and tyrosine phosphatase activity in WT and *Shp1*-Tg samples. **a***Shp1* gene expression was determined by RT-qPCR from spleen samples of 8–12-week-old female mice of each genotype. **b** Tyrosine phosphatase activity was measured in spleen samples of mice after SHP-1 immunoprecipitation. **c** SHP-1 protein expression was detected by Western blot in the spleen and bone marrow samples (mean ± SEM in panel **a**, % of WT in Fig. 1b, *n* = 3–7 mice/group, *p < 0.05, **p < 0.01, ***p < 0.001; ****p < 0.0001; one-way ANOVA)

We compared the expression and phosphatase function of SHP-1 in *Shp1*-Tg^+/+^ and *Shp1*-Tg^+/−^ mice relative to WT animals as shown in Fig. 1. *Shp1* gene expression in the spleen proved to be over 11-fold higher in homozygous *Shp1*-Tg^+/+^ mice compared to WT animals. *Shp1* expression in heterozygous (*Shp1*-Tg^+/−^) mice showed approximately 6-fold increase compared to WT mice. The extent of *Shp1* overexpression in homozygous and heterozygous transgenic mice was significantly different (Fig. 1a). The variation in expression among individual mice of the same strain is illustrated in Additional file 1. To test whether the increased expression of SHP-1 led to an increase in tyrosine phosphatase function, we immunoprecipitated SHP-1 from spleen cells of WT and transgenic mice and performed tyrosine phosphatase assays. Considering the enzyme activity of WT cells as 100%, tyrosine phosphatase activity in cells of *Shp1*-Tg^+/+^ mice was around 7-times higher, while around 3.7-times higher in samples of *Shp1*-Tg^+/−^ mice (Fig. 1b). SHP-1 protein expression was verified in spleen cell and bone marrow lysates of WT, homozygous, and heterozygous transgenic mice. Increased SHP-1 expression was detected in both the spleen and bone marrow of all transgenic mice compared to lysates from WT mice. GAPDH protein detection was used as a loading control (Fig. 1c). *Shp1* overexpression itself either in the homozygous or the heterozygous form did not appear to result in any adverse effect in vivo. Naïve *Shp1*-Tg mice looked healthy and did not differ in physical appearance from WT mice.

### B and T cell composition of the spleen in the presence of SHP-1 overexpression

We examined B and T cell subpopulations in the spleen of WT, *Shp1*-Tg^+/+^, and *Shp1*-Tg^+/−^ mice. Regarding T cells, we found significant differences between the WT and *Shp1*-Tg^+/+^ groups. The proportions of CD3+, CD4+, and CD8+ cells were significantly lower in *Shp1*-Tg^+/+^ mice than in WT animals. The proportion of total B220+ cells did not differ markedly between the genotypes. However, *Shp1*-Tg^+/+^ mice showed lower proportions of transitional 1 and higher proportions of transitional 2 B cells compared to WT cells. Marginal zone B cells appeared to be present in a higher proportion in the spleens of *Shp1*-Tg^+/−^ than in WT mice (Additional file 2).

### The effect of SHP-1 overexpression on the proliferation of T and B cells from naïve (non-immunized) WT and *Shp1*-Tg mice

**Fig. 2:**
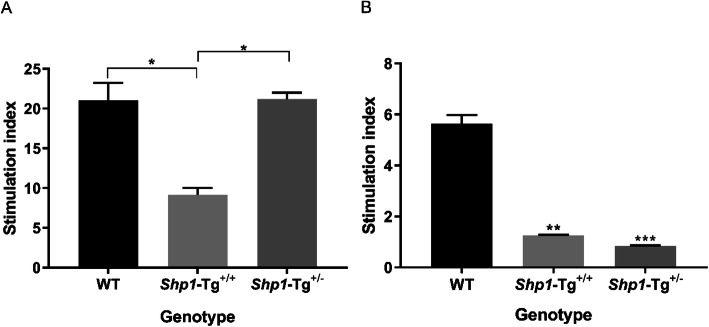
Proliferation of T and B cells from naïve WT and *Shp1*-Tg mice following polyclonal stimulation. **a** T cells and **b** B cells of the three genotypes of naïve mice were isolated from the spleen and stimulated with anti-CD3/CD28 or anti-IgM antibodies, respectively. Cell proliferation was measured by [^3^H] thymidine incorporation and expressed as stimulation index (mean ± SEM, *n* = 3/group, *p < 0.05, **p < 0.01, ***p < 0.001; one-way ANOVA)

In order to compare the magnitude of T and B cell proliferation in WT and *Shp1*-Tg mice, the spleen T and B cells of naïve WT, *Shp1*-Tg^+/+^ and *Shp1*-Tg^+/−^ mice were isolated and stimulated with anti-CD3/CD28 antibodies (for T cells) or anti-IgM antibody (for B cells). Cell proliferation was measured by [^3^H] thymidine incorporation. *Shp1*-Tg^+/−^ T cells responded significantly better to polyclonal stimulation than *Shp1*-Tg^+/+^ T cells, whose proliferation proved to be significantly lower than that of WT cells (Fig. 2a). B cells of naïve *Shp1*-Tg^+/−^ mice did not show a difference in proliferation compared to *Shp1*-Tg^+/+^ B cells, but cells from both Tg genotypes responded with significantly lower proliferation to anti-IgM stimulation than WT cells (Fig. 2b).

### Development of autoimmune arthritis in *Shp1*-Tg mice

**Fig. 3:**
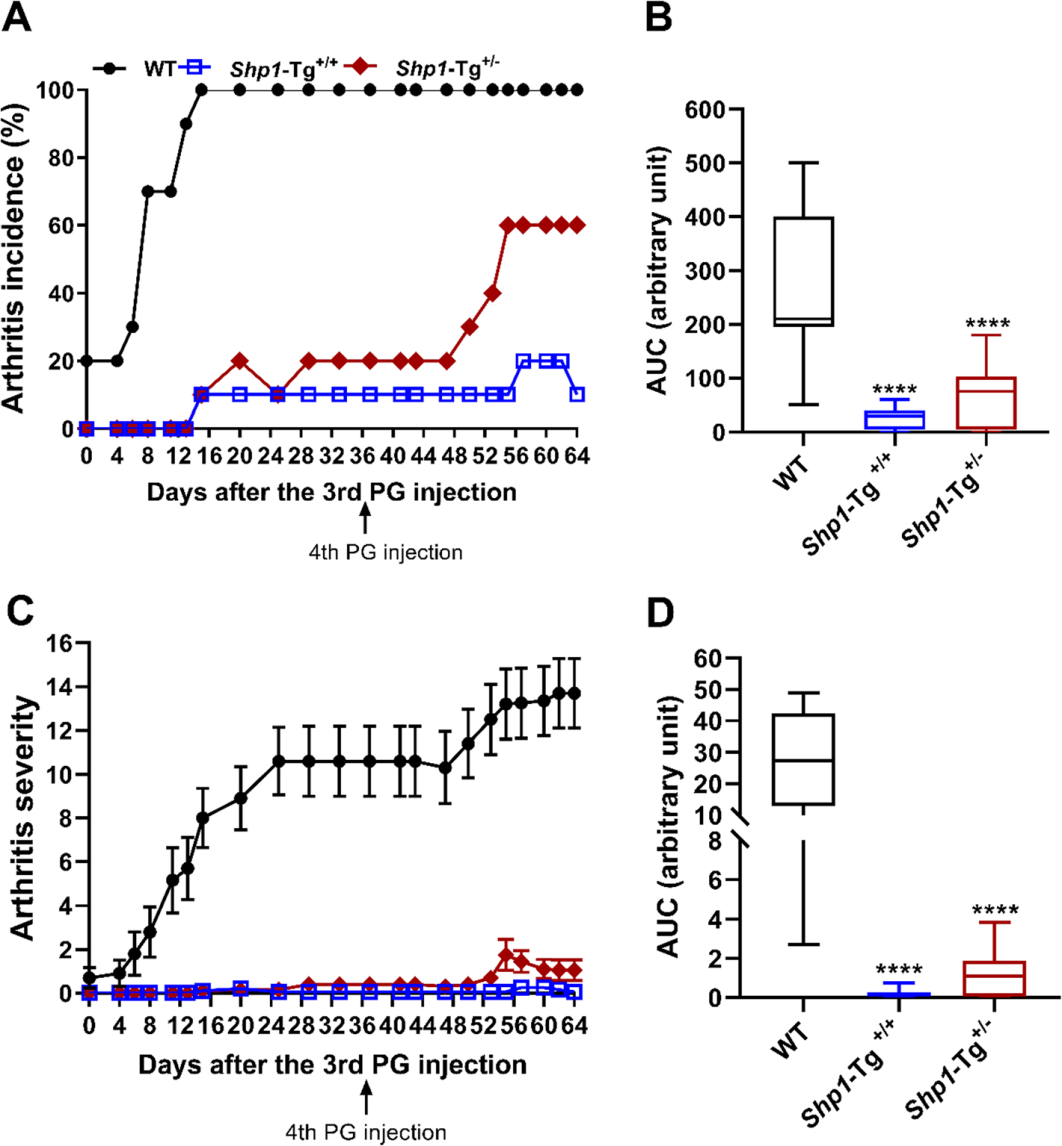
PGIA incidence and severity in WT, *Shp1*-Tg^+/+^, and *Shp1*-Tg^+/−^ mice. Mice were immunized 4 times with human PG then monitored and scored for symptoms of arthritis. **a** Arthritis incidence is expressed as the percent of arthritic mice of all immunized animals in each group. **b** Area under the curve (AUC) of arthritis incidence. **c** Arthritis severity is expressed as the mean visual arthritis score ± SEM. **d** AUC of arthritis severity (*n* = 10/group, ****p < 0.0001, WT vs the other groups; ANOVA followed by Dunn’s multiple comparison). AUCs are shown as box plots (center line, median; box limits, upper and lower quartiles; whiskers, maximu, and minimum values)

We induced PGIA in *Shp1*-Tg^+/−^ BALB/c mice to study the impact of moderate SHP-1 overexpression on arthritis susceptibility and antigen (PG)-specific adaptive immune responses. Our preliminary experiments indicated that *Shp1*-Tg^+/+^ mice were resistant to PGIA induction. WT, *Shp1*-Tg^+/+^ and *Shp1*-Tg^+/−^ mice (all in the PGIA susceptible BALB/c background) were immunized 4 times with human PG and signs of arthritis were visually scored 3 times a week. We found that while all of the WT mice developed arthritis, moderate overexpression of SHP-1 (as in *Shp1*-Tg^+/−^ mice) decreased arthritis incidence to around 60% relative to WT (Fig. 3a). AUC of arthritis incidence and severity in both *Shp1*-Tg^+/+^ and *Shp1*-Tg^+/−^ mice proved to be significantly lower compared to WT mice (Fig. 3b, d). Disease started at a much later time point in *Shp1*-Tg^+/−^ than in WT mice and remained very mild, usually with only one joint being affected. In accordance with our previous results, *Shp1*-Tg^+/+^ mice were essentially resistant to PGIA with minimal signs of arthritis compared to WT mice (Fig. 3c). We selected the heterozygous transgenic (*Shp1*-Tg^+/−^) line as a reference for further studies to assess the efficacy of therapeutic intervention in PGIA induced in WT mice.

### Antigen-induced cytokine secretion by spleen cells and serum anti-human PG antibody production in PG-immunized WT and *Shp1*-Tg mice

**Fig. 4:**
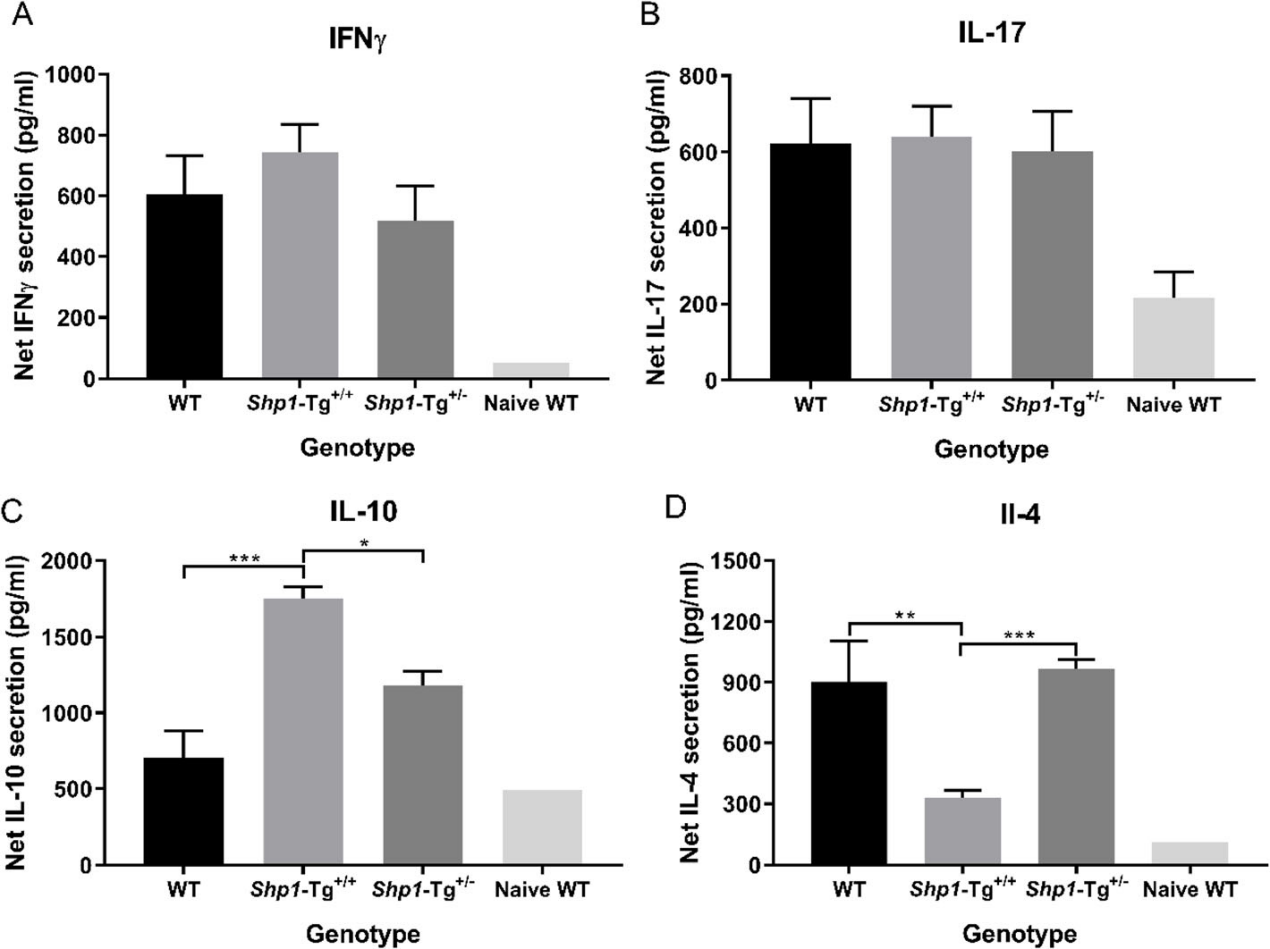
Net cytokine secretion by PG antigen-stimulated spleen cells from PG-immunized WT, *Shp1*-Tg^+/+^, and *Shp1*-Tg^+/−^ mice. **a** IFNγ, **b** IL-17, **c** IL-10, and **d** IL-4. Spleen cells of PG-immunized (or naïve WT) mice were stimulated with PG antigen in vitro for 4 days, then secreted cytokines were measured in the cell culture supernatants. Net cytokine secretion (pg/ml) is illustrated as stimulated minus non-stimulated values (mean ± SEM, *n* = 4–15/group, *p < 0.05, **p < 0.01, ***p < 0.001, one-way ANOVA)

Spleen cell cultures were set up from WT, *Shp1*-Tg^+/+^ and *Shp1*-Tg^+/−^ PGIA mice to determine the secreted cytokine concentrations in the supernatant after stimulation with PG. Net IFNγ, IL-17, IL-10, and IL-4 concentrations were calculated as stimulated minus non-stimulated values (in pg/ml). Results from naïve mice were included for comparison. We did not find significant differences between the 3 genotypes in IFNγ (Fig. 4a) or IL-17 (Fig. 4b) secretion by spleen cells. However, *Shp1*-Tg^+/+^ cells produced more IL-10 (Fig. 4c) and less IL-4 (Fig. 4d) than cells from WT animals. IL-10 secretion in *Shp1*-Tg^+/−^ mice was between the WT and *Shp1*-Tg^+/+^ values, but IL-4 secretion did not differ from that of WT animals. Interestingly, secretion of both IL-10 and IL-4 was significantly different in the cell cultures of heterozygous compared to homozygous transgenic animals. As shown in Fig. 4a–d, spleen cells from naïve WT mice responded with low cytokine secretion to PG stimulation, as expected.

Anti-human PG antibodies (IgG) were quantified in the sera of naïve and PG-immunized mice of the 3 genotypes (Additional file 3). In contrast with naïve animals, all PG-immunized mice mounted remarkable IgG antibody responses to human PG, without statistically significant differences among the WT, *Shp1*-Tg^+/+^, or *Shp1*-Tg^+/−^ groups.

### Differentially tyrosine-phosphorylated proteins in T cells from PG-immunized WT and *Shp1*-Tg mice

**Fig. 5:**
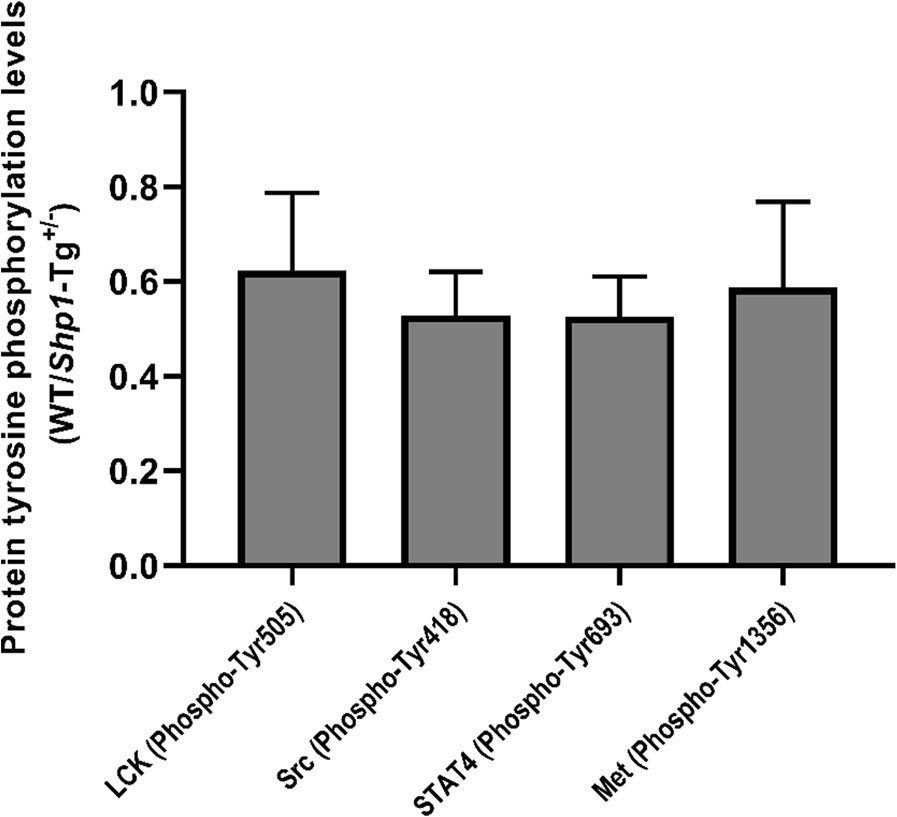
Differentially expressed tyrosine phosphorylated proteins in CD4^+^CD25^−^ spleen cells from PG-immunized WT and *Shp1*-Tg^+/−^ mice. Protein tyrosine phosphorylation levels are expressed as *Shp1*-Tg^+/−^ vs WT ratios of the most differentially tyrosine phosphorylated proteins (mean ± SEM, *n* = 3 experiments, Mann-Whitney test did not detect significant differences between the samples of *Shp1*-Tg^+/−^ and WT mice)

It is known that both RA and PGIA are T cell-driven autoimmune diseases and that SHP-1 plays an inhibitory role in TCR signal transduction. Our results showed that overexpression of SHP-1 led to decreased proliferation of CD4^+^ T cells in mice. Therefore, we sought to determine which phosphoproteins were differentially phosphorylated (i.e., underphosphorylated) at tyrosine residues in T cells of *Shp1*-Tg^+/−^ mice as compared to T cells of WT animals. Tyrosine Phosphorylation ProArray, using 228 site-specific anti-phospho-tyrosine antibodies, was performed on spleen T cell extracts from the 2 genotypes of PG-immunized mice. After summarizing data from the 3 biological replicates, 9 sites showed > 20% decrease in phosphorylation levels, and 5 sites showed increases of > 20%. The changes of phosphorylation levels between WT and Shp1-Tg samples at the other 214 sites were less than 20%. We focused on sites with decreased tyrosine phosphorylation considering the enhanced tyrosine phosphatase activity in Shp1-Tg^+/−^ mice. Although we did not detect statistically significant differences (possibly due to the limited number of biological replicates), we identified 4 phosphoproteins that showed the most profound reduction of site-specific tyrosine phosphorylation in T cells of *Shp1*-Tg^+/−^ mice as compared to WT T cells (both from PG-immunized animals). In the order of underphosphorylation levels, these phosphoproteins were as follows: Src (Phospho-Tyr418) 47% decrease, STAT4 (Phospho-Tyr693) 47% decrease, Met (Phospho-Tyr1356) 41% decrease, and Lck (Phospho-Tyr505) 38% decrease (Fig. 5). Panther pathway analysis annotated the JAK/STAT signaling pathway to these phosphoproteins when compared to the genes of proteins included in the microarray; however, the overrepresentation test did not reach statistical power possibly due to the small number of reference genes (Panther pathways analysis, *p* = 0.273). We found relative minor changes in the tyrosine phosphorylation levels of known targets of SHP-1 such as Lyn (11% decrease), and Syk and Zap70 (0–6% and 2–5% change ranges, respectively).

### Reduced arthritis severity and incidence after SHP-1 activator treatment of WT mice

PGIA was induced in WT female BALB/c mice. We applied regorafenib daily at 10 or 20 mg/kg dose per group via oral gavage starting on the day of the third PG injection. PGIA incidence and severity, in vitro cytokine secretion, and the presence of serum anti-human PG IgG antibodies were evaluated. *Shp1*-Tg^+/−^ mice immunized with PG but without activator treatment were included as additional reference controls.

**Fig. 6:**
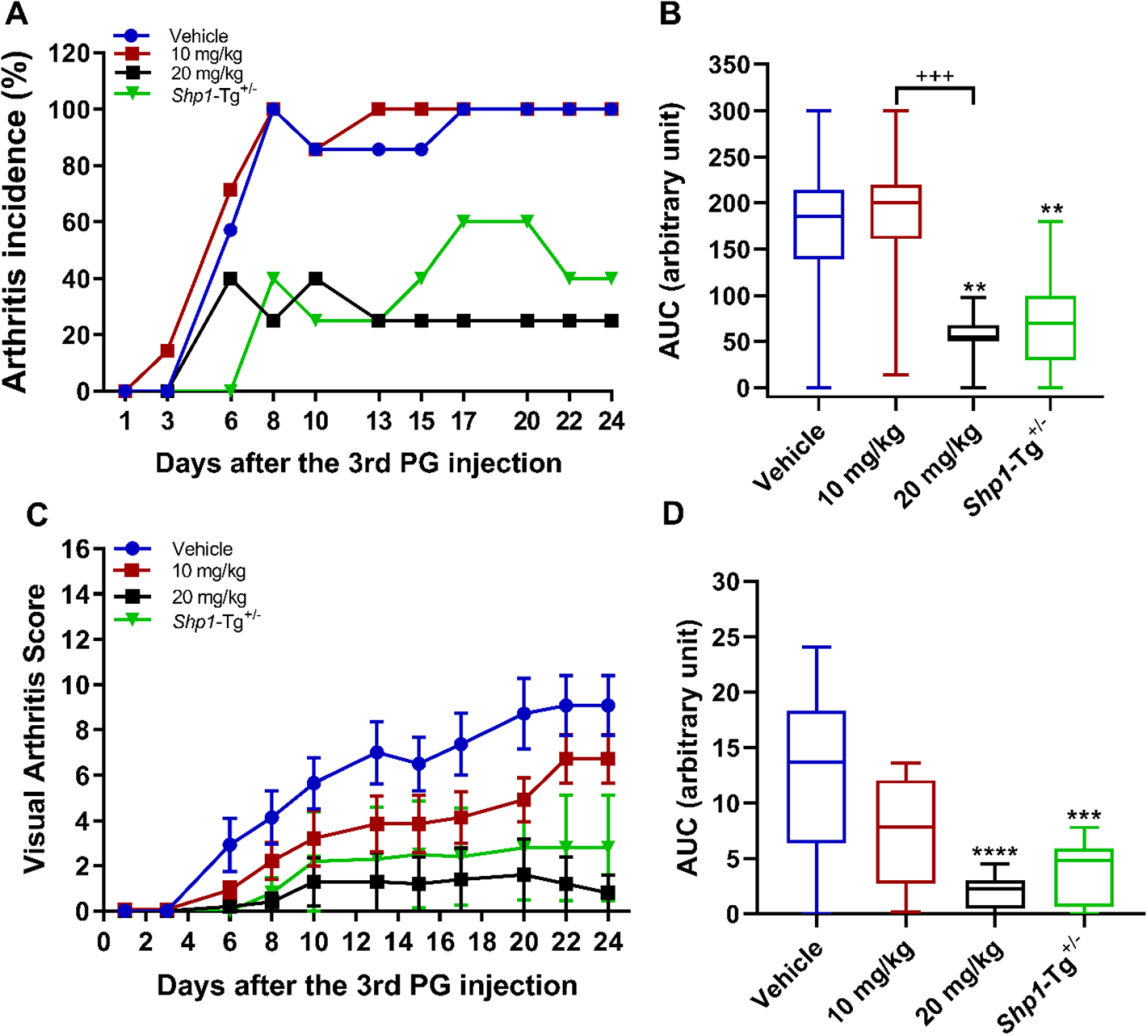
PGIA incidence and severity in WT mice after SHP-1 activator administration. Female BALB/c mice were immunized with human PG. Regorafenib was administered daily via oral gavage from the day of the third PG injection. Mice were monitored and scored for symptoms of arthritis 3 times a week. **a** Arthritis incidence is expressed as the percent of arthritic mice of all animals in each group. **b** AUC of arthritis incidence. **c** Arthritis severity is expressed as the mean visual arthritis score ± SEM. **d** AUC of arthritis severity (*n* = 5–7/group, **p < 0.01, ***p < 0.001, ****p < 0.0001 vehicle-treated vs 20 mg/kg and *Shp1*-Tg^+/−^ groups; ^+++^*p* < 0.001 10 mg/kg vs 20 mg/kg regorafenib; ANOVA followed by Tukey’s multiple comparison). AUCs are shown as box plots (center line, median; box limits, upper and lower quartiles; whiskers, maximum and minimum values)

**Fig. 7:**
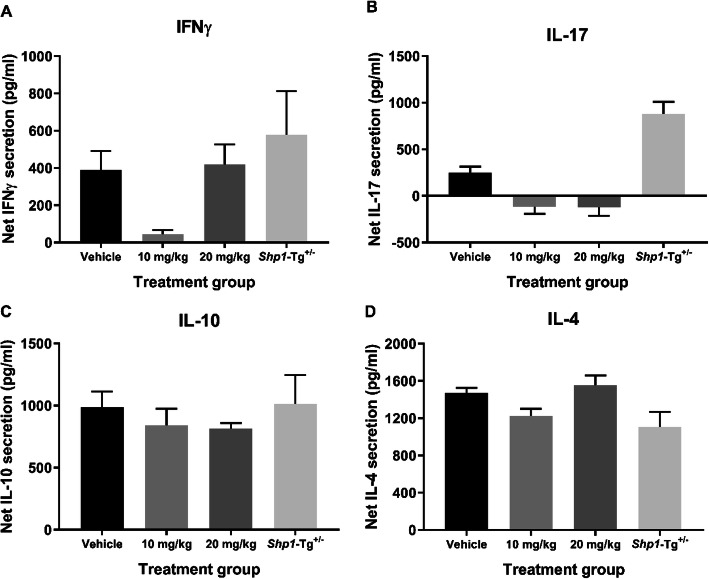
Net cytokine secretion in regorafenib treated WT or *Shp1*-Tg^+/−^ PGIA mice. **a** IFNγ, **b** IL-17, **c** IL-10, and **d** IL-4. Spleen cells of PGIA mice were stimulated with PG in vitro for 4 days then secreted cytokines were measured in the cell culture supernatants. Net cytokine secretion (pg/ml) is illustrated as stimulated minus non-stimulated values (mean ± SEM, *n* = 5/group, one-way ANOVA)

Daily administration of 20 mg/kg regorafenib significantly reduced PGIA incidence and severity compared to vehicle (Fig. 6). These results were similar to disease incidence and severity in PG-immunized *Shp1*-Tg^+/−^ mice without regorafenib treatment. However, 20 mg/kg regorafenib resulted in weight loss and increased mortality rate, presumably due to either robust immune suppression or regorafenib toxicity, or both. In order to determine the effect of regorafenib on net cytokine secretion in cell culture supernatant, spleen cells were stimulated with PG in vitro (Fig. 7a–d). We did not find statistically significant differences in IFNγ, IL-17, IL-10, or IL-4 secretion, although IFNγ production was strongly suppressed in the 10 mg/kg treatment group. Net IL-17 secretion seemed to be overall poor in response to PG. We also tested the anti-human PG antibody content in the sera of regorafenib treated WT and *Shp1*-Tg^+/−^ mice (Additional file 4). There was no difference between the treatment groups.

**Fig. 8:**
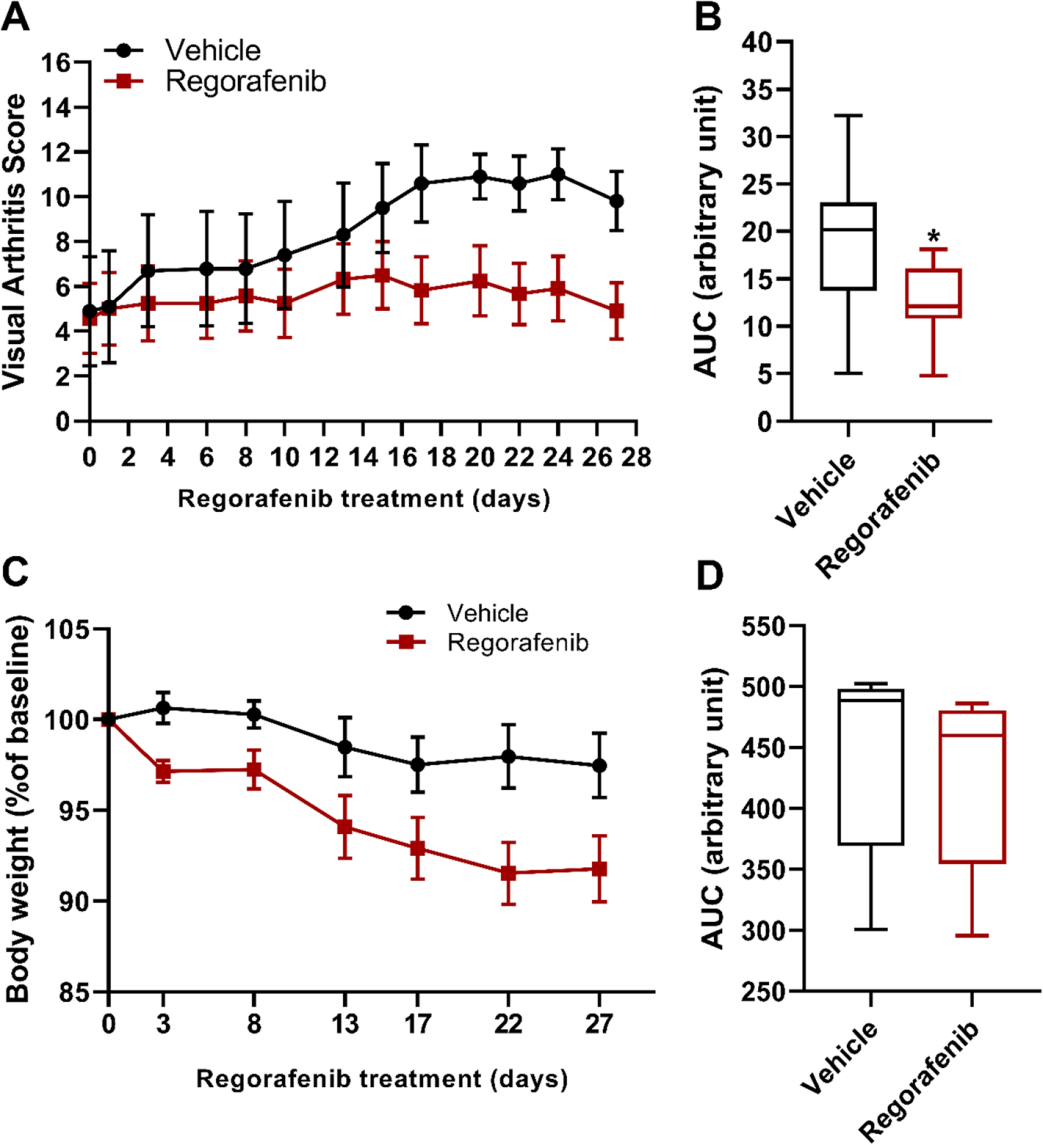
PGIA severity and body weight of WT mice after therapeutic regorafenib administration. Mice were immunized with human PG to induce PGIA. Regorafenib or vehicle was administered 5 times a week via oral gavage after the initial signs of arthritis developed. **a** Mice were monitored and scored for symptoms of arthritis 3 times a week. **b** AUC of visual arthritis score. **c** Changes in body weight, calculated as the % of original body weight for each mouse, measured 2 times a week. **d** AUC of body weight. Values are illustrated as the mean ± SEM or as the percent of baseline (*n* = 9–10/group, *p < 0.05 vehicle-treated vs 15 mg/kg regorafenib groups; Student’s *t* test or Mann-Whitney test). AUCs are shown as box plots (center line, median; box limits, upper and lower quartiles; whiskers, maximum and minimum values)

We also sought to explore if SHP-1 activator administration could ameliorate arthritis when started after the development of the initial signs of arthritis (therapeutic treatment). WT mice were immunized with PG as described. When the average visual arthritis score reached approximately 3, mice were divided into 2 groups with similar initial disease scores. Regorafenib at 15 mg/kg or vehicle was administered via oral gavage 5 times a week and body weights were monitored. Regorafenib treatment started after the initial symptoms of arthritis significantly decreased arthritis severity as indicated by the lower VAS compared to vehicle (Fig. 8a, b). There was a slight decrease in the body weight of mice in the activator-treated group (less than 10% of the baseline), but no significant difference was observed between the two groups (Fig. 8c, d).

## Discussion

SHP-1 is an important negative regulator of lymphocyte signaling [33]. Its loss-of-function mutation results in inflammation and autoimmunity, but without joint involvement [16, 19]. Since *Shp1* deficiency leads to the motheaten phenotype and multiple autoimmune features, we wanted to determine if *Shp1* overexpression has any effect on arthritis (PGIA) incidence and/or severity. This idea was based on the facts that autoimmunity plays a central role in PGIA and that many signaling proteins within immune cells involved in joint inflammation are under the control of SHP-1. We found that homozygous transgenic (*Shp1*-Tg^+/+^) mice were resistant to PGIA, but heterozygous transgenic (*Shp1*-Tg^+/−^) mice displaying moderate SHP-1 overexpression were not completely resistant to the disease. The complete resistance of the homozygous *Shp1*-Tg mice to PGIA limited the implication of the homozygous model in arthritis. Thus, we selected heterozygous *Shp1*-Tg^+/−^ BALB/c mice for further analysis as they were more suitable than their homozygous counterparts for investigating the role of SHP-1 in autoimmune arthritis.

We found that polyclonally activated T and B cells of naïve *Shp1*-Tg^+/+^ mice showed significantly lower proliferation compared to similar cells of WT mice. In naïve *Shp1*-Tg^+/−^ mice, the extent of polyclonal T cell proliferation did not differ significantly from that in WT animals, but proliferation was very low in cultures of activated B cells from naïve *Shp1*-Tg^+/−^ mice. It is likely that anti-CD3/CD28 stimulation induced a stronger response in T cells than anti-IgM did in B cells. Therefore, *Shp1* overexpression had a lower inhibitory (counter) effect on T cells than on B cells.

In PG-immunized *Shp1*-Tg^+/−^ mice, antibody production against the immunizing antigen did not differ from the production by WT animals as seen by comparable anti-human PG antibody levels in the sera. SHP-1 has been shown to play a crucial role in T cell signaling and acts as a regulator of T cell susceptibility to Treg mediated suppression [34]. Tyrosine phosphorylation profiling of CD4^+^CD25^−^ T cells from PG-immunized mice showed differences between *Shp1*-Tg^+/−^ and WT animals in the phosphorylation of Src (Phospho-Tyr418), STAT4 (Phospho-Tyr693), Met (Phospho-Tyr1356), and Lck (Phospho-Tyr505), suggesting the involvement of the JAK/STAT signal transduction pathway in regulating T cell responses under the condition of increased phosphatase activity (i.e., decreased protein tyrosine phosphorylation).

The JAK/STAT pathway plays a major role in cytokine receptor signaling [35]. The involvement of SHP-1 in the α/β interferon-stimulated JAK/STAT pathway has been described using macrophages from motheaten mice [36]. Tyrosine phosphorylation of several signaling proteins was reduced in SHP-1 overexpressing mice, suggesting that SHP-1 is a crucial regulator of this pathway. It has been shown that STAT-regulated gene expression is increased in RA synovial tissue [37], and STAT3 is constitutively activated in RA [38]. The recently developed JAK inhibitors for the treatment of RA prevent signal transduction induced by pro-inflammatory cytokines, indicating an important role of the JAK pathway in this disease. Additionally, JAK inhibitors are orally available, unlike biologics that act outside the cells and need to be administered parenterally. In light of this, our finding that the most downregulated Tyr phosphorylated proteins in the presence of SHP-1 overproduction are associated with the JAK/STAT pathway are particularly interesting. SHP-1 overproduction might lead to the suppression of pro-inflammatory gene expression via inactivation of STAT transcription factors, thus disrupting signal transduction at early checkpoints.

STAT4 has a regulatory function in T helper 1 (Th1) immune responses [39]. It has been shown that STAT4 phosphorylation both on tyrosine and serine residues is important for IFNγ secretion [40]. It has been found in B cells that STAT activation can occur in a JAK-independent route through Lyn, which directly phosphorylates STAT3 [41].

Pharmacological activation of the SHP-1 phosphatase activity has been shown to induce apoptosis in cancer cells [42]. Regorafenib (sometimes referred to as a “multi kinase inhibitor”) has been reported to activate SHP-1 by relieving its auto inhibition and exerts a tumor suppressive effect [15]. Regorafenib was approved by the FDA in 2012 for the treatment of metastatic colorectal cancer. To our knowledge, there are no published data for the effect of regorafenib on autoimmune arthritis. We tested 2 different dosing schedules and 3 doses of this SHP-1 activator in PG-immunized WT mice. Treatment with daily 20 mg/kg per os after the 3rd PG immunization prevented the development of PGIA in the majority of mice. However, we observed potentially toxic effect appearing as weight loss and increased mortality rate. Therefore, we decided to conduct the next therapeutic regorafenib treatment study by administering 15 mg/kg 5 times a week and monitoring body weight and general wellness. Therapeutic regorafenib administration resulted in decreases in arthritis severity compared to vehicle treatment, likely due to SHP-1 activation. However, as an SHP-1 activator, regorafenib can indirectly act on multiple kinases and might also enhance the activity of other SH2 domain-containing phosphatases; hence, the specificity of the drug’s effect is not certain. We hypothesize that inhibition of the JAK/STAT pathway might be an important aspect of arthritis suppression in the presence of moderate SHP-1 overproduction. However, caution must be applied when assessing the therapeutic potential of tyrosine phosphatase activation for autoimmune arthritis. Inhibition of the JAK/STAT signaling can result in non-selective inhibition of anti-inflammatory cytokines as well as general immunosuppression. As many signaling pathways are inter-related and SHP-1 has diverse effects on multiple cell types, a better understanding of SHP-1 activity and application of more selective phosphatase activators are necessary for future development of SHP-1-targeting drugs.

## Conclusions

We demonstrated that *Shp1*-Tg^+/+^ mice are completely resistant to the development of PGIA due to robust SHP-1 overexpression. Partially, arthritis-susceptible *Shp1*-Tg^+/−^ mice are useful for investigating the role of SHP-1 in autoimmune arthritis such as PGIA. Our results show that regorafenib treatment prevents the development of PGIA in WT mice as well as reduces arthritis severity when administered after the onset of the disease, possibly through pharmacological activation of SHP-1 tyrosine phosphatase. We also demonstrated that overexpression of SHP-1 in T cells of PG-immunized mice decreases the tyrosine phosphorylation of important signaling proteins in the JAK/STAT pathway.

## Supplementary information

**Additional file 1 ***Shp1* gene expression in WT and *Shp1*-Tg samples. *Shp1* gene expression was determined by RT-qPCR from spleen samples of 8-12 weeks old female mice of each genotype (mean ±SEM, *n*=7/group, **p<0.01, ***p<0.001; ****p<0.0001; one-way ANOVA).

**Additional file 2 **Spleen cell populations of WT, Shp1-Tg+/+ and Shp1-Tg+/- mice. CD3+ cells are shown as % of lymphocytes. CD4+ and CD8+ cells are shown as % of CD3+ cells. Total B220+ cells are illustrated as the % of lymphocytes. Marginal zone, transitional 1, transitional 2 and follicular B cells are illustrated as % of B220+ cells. Plasma cells are seen as % of lymphocytes. Red color indicates statistically significant difference (*n*=4/group, Kruskal-Wallis test). B cell subpopulations were defined as: marginal zone: IgM high, CD21 high, CD23-; transitional 1: IgM high, CD21 low, CD23-; transitional 2: IgM high, CD21 med, CD23+; follicular: IgM low, CD21 med, CD23+; plasma cells: B220 low, CD19-, CD138+.

**Additional file 3 **Anti-human PG antibody levels in the sera of naïve and PG-immunized mice. Anti-human PG IgG antibody contents were measured in the sera of naïve and PG-immunized WT and the 2 genotypes of *Shp1*-Tg mice and expressed as optical density at 450 nm (mean±SEM, *n*=5-10/group, one-way ANOVA).

**Additional file 4.** Serum anti-human PG antibody levels in PG-immunized and SHP-1 activator-treated or untreated mice. Anti-human PG antibody content was measured in the sera of PG-immunized mice. WT mice underwent vehicle or regorafenib treatment, while Shp1-Tg^+/-^ mice did not receive treatment. Serum antibody titers are expressed as optical density (mean±SEM, n=5/group, one-way ANOVA).

## Data Availability

The datasets used and/or analyzed for the current study are available from the corresponding author on reasonable request.

## Abbreviations

Ab: Antibody
autoAbs: Autoantibodies
BCR: B cell receptor
cpm: Counts per minute
DMEM: Dulbecco’s modified Eagle’s medium
ELISA: Enzyme-linked immunosorbent assay
FBS: Fetal bovine serum
IFNγ: Interferon gamma
IL-4: Interleukin-4
IL-10: Interleukin-10
IL-17: Interleukin-17
ip.: Intraperitoneal
ITAM: Immunoreceptor tyrosine-based activation motif
ITIM: Immunoreceptor tyrosine-based inhibition motif
me: Motheaten
OD: Optical density
PBS: Phosphate-buffered saline
PCR: Polymerase chain reaction
PG: Proteoglycan (aggrecan)
PGIA: PG-induced arthritis
p.os: Per os
RA: Rheumatoid arthritis
rhG1: Recombinant G1 domain of human PG
RT qPCR: Real-time quantitative PCR
SEM: Standard error of the mean
SHP-1: Src homology region 2 domain-containing phosphatase-1
SI: Stimulation index
TCR: T cell receptor
Tg: Transgenic
Th: T helper
Treg: T regulatory
VAS: Visual arthritis score
WT: Wild type

## References

[1] Garg M, Wahid M, Khan FD (2020). Regulation of peripheral and central immunity: understanding the role of Src homology 2 domain-containing tyrosine phosphatases, SHP-1 & SHP-2. Immunobiology..

[2] Daeron M, Jaeger S, Du Pasquier L, Vivier E (2008). Immunoreceptor tyrosine-based inhibition motifs: a quest in the past and future. Immunol Rev.

[3] Mustelin T, Tasken K (2003). Positive and negative regulation of T-cell activation through kinases and phosphatases. Biochem J.

[4] Pani G, Kozlowski M, Cambier JC, Mills GB, Siminovitch KA (1995). Identification of the tyrosine phosphatase PTP1C as a B cell antigen receptor-associated protein involved in the regulation of B cell signaling. J Exp Med.

[5] Pani G, Fischer KD, Mlinaric-Rascan I, Siminovitch KA (1996). Signaling capacity of the T cell antigen receptor is negatively regulated by the PTP1C tyrosine phosphatase. J Exp Med.

[6] Tamir I, Dal Porto JM, Cambier JC (2000). Cytoplasmic protein tyrosine phosphatases SHP-1 and SHP-2: regulators of B cell signal transduction. Curr Opin Immunol.

[7] Raab M, Rudd CE (1996). Hematopoietic cell phosphatase (HCP) regulates p56LCK phosphorylation and ZAP-70 binding to T cell receptor zeta chain. Biochem Biophys Res Commun.

[8] Chiang GG, Sefton BM (2001). Specific dephosphorylation of the Lck tyrosine protein kinase at Tyr-394 by the SHP-1 protein-tyrosine phosphatase. J Biol Chem.

[9] Au-Yeung BB, Deindl S, Hsu LY, Palacios EH, Levin SE, Kuriyan J (2009). The structure, regulation, and function of ZAP-70. Immunol Rev.

[10] Nishizumi H, Horikawa K, Mlinaric-Rascan I, Yamamoto T (1998). A double-edged kinase Lyn: a positive and negative regulator for antigen receptor-mediated signals. J Exp Med.

[11] Xu Y, Harder KW, Huntington ND, Hibbs ML, Tarlinton DM (2005). Lyn tyrosine kinase: accentuating the positive and the negative. Immunity..

[12] Dustin LB, Plas DR, Wong J, Hu YT, Soto C, Chan AC (1999). Expression of dominant-negative src-homology domain 2-containing protein tyrosine phosphatase-1 results in increased Syk tyrosine kinase activity and B cell activation. J Immunol.

[13] Coggeshall KM, Nakamura K, Phee H (2002). How do inhibitory phosphatases work?. Mol Immunol.

[14] Martin A, Tsui HW, Shulman MJ, Isenman D, Tsui FW (1999). Murine SHP-1 splice variants with altered Src homology 2 (SH2) domains. Implications for the SH2-mediated intramolecular regulation of SHP-1. J Biol Chem.

[15] Fan LC, Teng HW, Shiau CW, Lin H, Hung MH, Chen YL (2014). SHP-1 is a target of regorafenib in colorectal cancer. Oncotarget..

[16] Tsui HW, Siminovitch KA, de Souza L, Tsui FW (1993). Motheaten and viable motheaten mice have mutations in the haematopoietic cell phosphatase gene. Nat Genet.

[17] Nesterovitch AB, Gyorfy Z, Hoffman MD, Moore EC, Elbuluk N, Tryniszewska B (2011). Alteration in the gene encoding protein tyrosine phosphatase nonreceptor type 6 (PTPN6/SHP1) may contribute to neutrophilic dermatoses. Am J Pathol.

[18] Speir M, Nowell CJ, Chen AA, O'Donnell JA, Shamie IS, Lakin PR (2020). Ptpn6 inhibits caspase-8- and Ripk3/Mlkl-dependent inflammation. Nat Immunol.

[19] Nesterovitch AB, Szanto S, Gonda A, Bardos T, Kis-Toth K, Adarichev VA (2011). Spontaneous insertion of a b2 element in the ptpn6 gene drives a systemic autoinflammatory disease in mice resembling neutrophilic dermatosis in humans. Am J Pathol.

[20] Mauldin IS, Tung KS, Lorenz UM (2012). The tyrosine phosphatase SHP-1 dampens murine Th17 development. Blood..

[21] Pao LI, Lam KP, Henderson JM, Kutok JL, Alimzhanov M, Nitschke L (2007). B cell-specific deletion of protein-tyrosine phosphatase Shp1 promotes B-1a cell development and causes systemic autoimmunity. Immunity..

[22] Abram CL, Lowell CA (2017). Shp1 function in myeloid cells. J Leukoc Biol.

[23] Glant TT, Mikecz K, Arzoumanian A, Poole AR (1987). Proteoglycan-induced arthritis in BALB/c mice. Clinical features and histopathology. Arthritis Rheum.

[24] Mikecz K, Glant TT, Poole AR (1987). Immunity to cartilage proteoglycans in BALB/c mice with progressive polyarthritis and ankylosing spondylitis induced by injection of human cartilage proteoglycan. Arthritis Rheum.

[25] Glant TT, Finnegan A, Mikecz K (2003). Proteoglycan-induced arthritis: immune regulation, cellular mechanisms, and genetics. Crit Rev Immunol.

[26] Glant TT, Radacs M, Nagyeri G, Olasz K, Laszlo A, Boldizsar F (2011). Proteoglycan-induced arthritis and recombinant human proteoglycan aggrecan G1 domain-induced arthritis in BALB/c mice resembling two subtypes of rheumatoid arthritis. Arthritis Rheum.

[27] Firestein GS (2005). Immunologic mechanisms in the pathogenesis of rheumatoid arthritis. J Clin Rheumatol.

[28] Markovics A, Ocsko T, Katz RS, Buzas EI, Glant TT, Mikecz K (2016). Immune recognition of citrullinated proteoglycan aggrecan epitopes in mice with proteoglycan-induced arthritis and in patients with rheumatoid arthritis. PLoS One.

[29] Hanyecz A, Berlo SE, Szanto S, Broeren CP, Mikecz K, Glant TT (2004). Achievement of a synergistic adjuvant effect on arthritis induction by activation of innate immunity and forcing the immune response toward the Th1 phenotype. Arthritis Rheum.

[30] Kurko J, Vida A, Ocsko T, Tryniszewska B, Rauch TA, Glant TT (2014). Suppression of proteoglycan-induced autoimmune arthritis by myeloid-derived suppressor cells generated in vitro from murine bone marrow. PLoS One.

[31] Glant TT, Ocsko T, Markovics A, Szekanecz Z, Katz RS, Rauch TA (2016). Characterization and localization of citrullinated proteoglycan aggrecan in human articular cartilage. PLoS One.

[32] Wilhelm SM, Dumas J, Adnane L, Lynch M, Carter CA, Schutz G (2011). Regorafenib (BAY 73-4506): a new oral multikinase inhibitor of angiogenic, stromal and oncogenic receptor tyrosine kinases with potent preclinical antitumor activity. Int J Cancer.

[33] Neel BG, Tonks NK (1997). Protein tyrosine phosphatases in signal transduction. Curr Opin Cell Biol.

[34] Mercadante ER, Lorenz UM (2017). T cells deficient in the tyrosine phosphatase SHP-1 resist suppression by regulatory T cells. J Immunol.

[35] Liu KD, Gaffen SL, Goldsmith MA (1998). JAK/STAT signaling by cytokine receptors. Curr Opin Immunol.

[36] David M, Chen HE, Goelz S, Larner AC, Neel BG (1995). Differential regulation of the alpha/beta interferon-stimulated Jak/Stat pathway by the SH2 domain-containing tyrosine phosphatase SHPTP1. Mol Cell Biol.

[37] van der Pouw Kraan TC, van Gaalen FA, Kasperkovitz PV, Verbeet NL, Smeets TJ, Kraan MC (2003). Rheumatoid arthritis is a heterogeneous disease: evidence for differences in the activation of the STAT-1 pathway between rheumatoid tissues. Arthritis Rheum.

[38] Ivashkiv LB (1996). Cytokine expression and cell activation in inflammatory arthritis. Adv Immunol.

[39] Thieu VT, Yu Q, Chang HC, Yeh N, Nguyen ET, Sehra S (2008). Signal transducer and activator of transcription 4 is required for the transcription factor T-bet to promote T helper 1 cell-fate determination. Immunity..

[40] Morinobu A, Gadina M, Strober W, Visconti R, Fornace A, Montagna C (2002). STAT4 serine phosphorylation is critical for IL-12-induced IFN-gamma production but not for cell proliferation. Proc Natl Acad Sci U S A.

[41] Wang L, Kurosaki T, Corey SJ (2007). Engagement of the B-cell antigen receptor activates STAT through Lyn in a Jak-independent pathway. Oncogene..

[42] Liu CY, Huang TT, Chu PY, Huang CT, Lee CH, Wang WL (2017). The tyrosine kinase inhibitor nintedanib activates SHP-1 and induces apoptosis in triple-negative breast cancer cells. Exp Mol Med.

