# Supplementary material for: Regulation of autoimmune arthritis by the SHP-1 tyrosine phosphatase
Source: Arthritis Res Ther. 2020 Jun 26;22:160. doi: 10.1186/s13075-020-02250-8 (PMC7318740; doi:10.1186/s13075-020-02250-8)
Supplement: Supplementary file 1 — Additional file 1 Shp1 gene expression in WT and Shp1-Tg samples. Shp1 gene expression was determined by RT-qPCR from spleen samples of 8-12 weeks old female mice of each genotype (mean ±SEM, n=7/group, **p<0.01, ***p<0.001; ****p<0.0001; one-way ANOVA). [file 13075_2020_2250_MOESM1_ESM.docx]

ADDITIONAL FILE 1


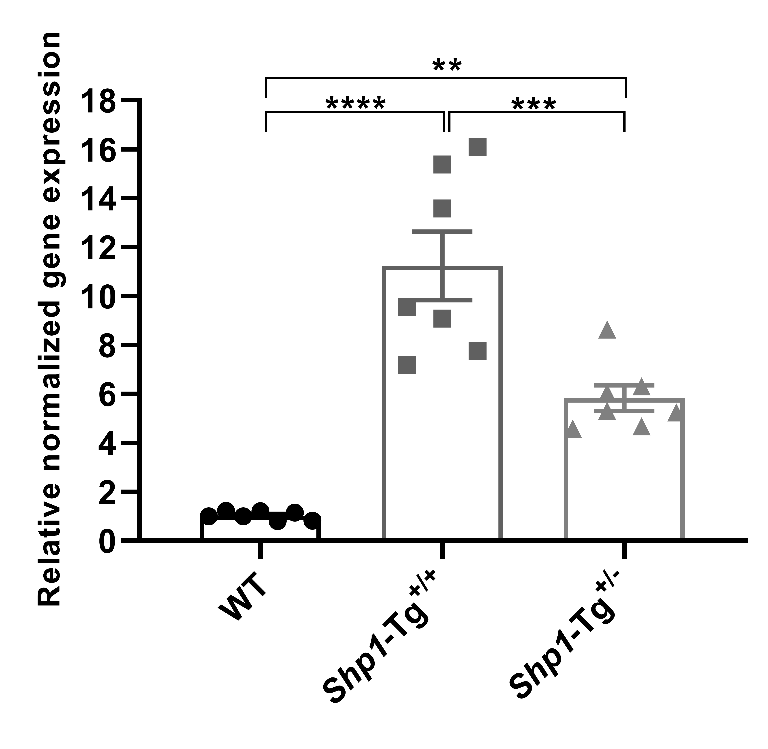


**Additional file 1. *Shp1* gene expression in WT and *Shp1*-Tg samples.** *Shp1* gene expression was determined by RT-qPCR from spleen samples of 8-12 weeks old female mice of each genotype (mean ±SEM, n=7/group, **p<0.01, ***p<0.001; ****p<0.0001; one-way ANOVA).
