# Supplementary material for: Regulation of autoimmune arthritis by the SHP-1 tyrosine phosphatase
Source: Arthritis Res Ther. 2020 Jun 26;22:160. doi: 10.1186/s13075-020-02250-8 (PMC7318740; doi:10.1186/s13075-020-02250-8)
Supplement: Supplementary file 3 — Additional file 3 Anti-human PG antibody levels in the sera of naïve and PG-immunized mice. Anti-human PG IgG antibody contents were measured in the sera of naïve and PG-immunized WT and the 2 genotypes of Shp1-Tg mice and expressed as optical density at 450 nm (mean±SEM, n=5-10/group, one-way ANOVA). [file 13075_2020_2250_MOESM3_ESM.docx]

ADDITIONAL FILE 3


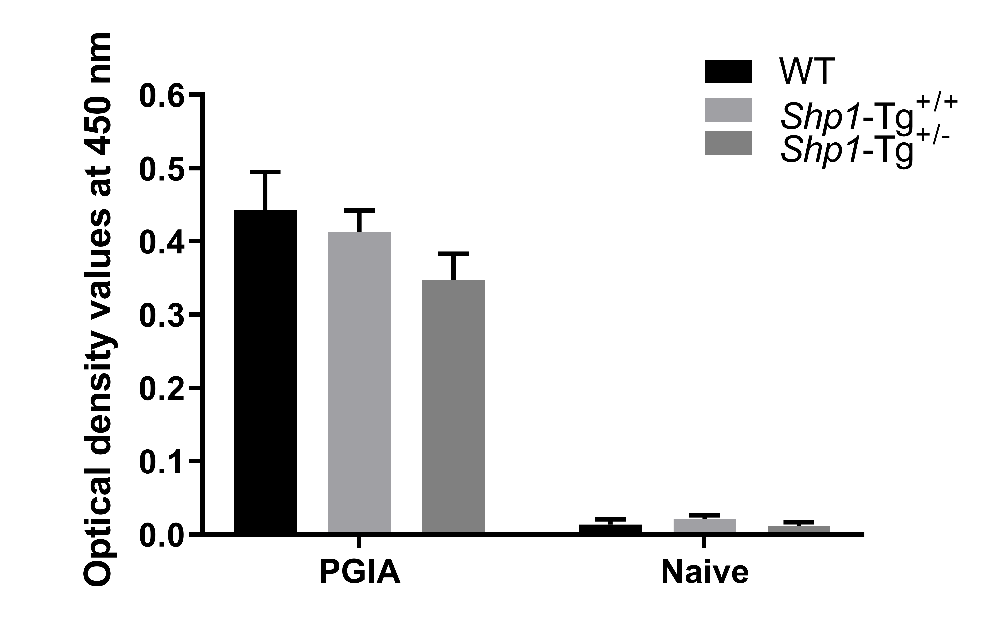


**Additional file 3. Anti-human PG antibody levels in the sera of naïve and PG-immunized mice.** Anti-human PG IgG antibody contents were measured in the sera of naïve and PG-immunized WT and the 2 genotypes of *Shp1*-Tg mice and expressed as optical density at 450 nm (mean±SEM, n=5-10/group, one-way ANOVA).
