# Supplementary material for: Regulation of autoimmune arthritis by the SHP-1 tyrosine phosphatase
Source: Arthritis Res Ther. 2020 Jun 26;22:160. doi: 10.1186/s13075-020-02250-8 (PMC7318740; doi:10.1186/s13075-020-02250-8)
Supplement: Supplementary file 4 — Additional file 4. Serum anti-human PG antibody levels in PG-immunized and SHP-1 activator-treated or untreated mice. Anti-human PG antibody content was measured in the sera of PG-immunized mice. WT mice underwent vehicle or regorafenib treatment, while Shp1-Tg+/- mice did not receive treatment. Serum antibody titers are expressed as optical density (mean±SEM, n=5/group, one-way ANOVA). [file 13075_2020_2250_MOESM4_ESM.docx]

ADDITIONAL FILE 4


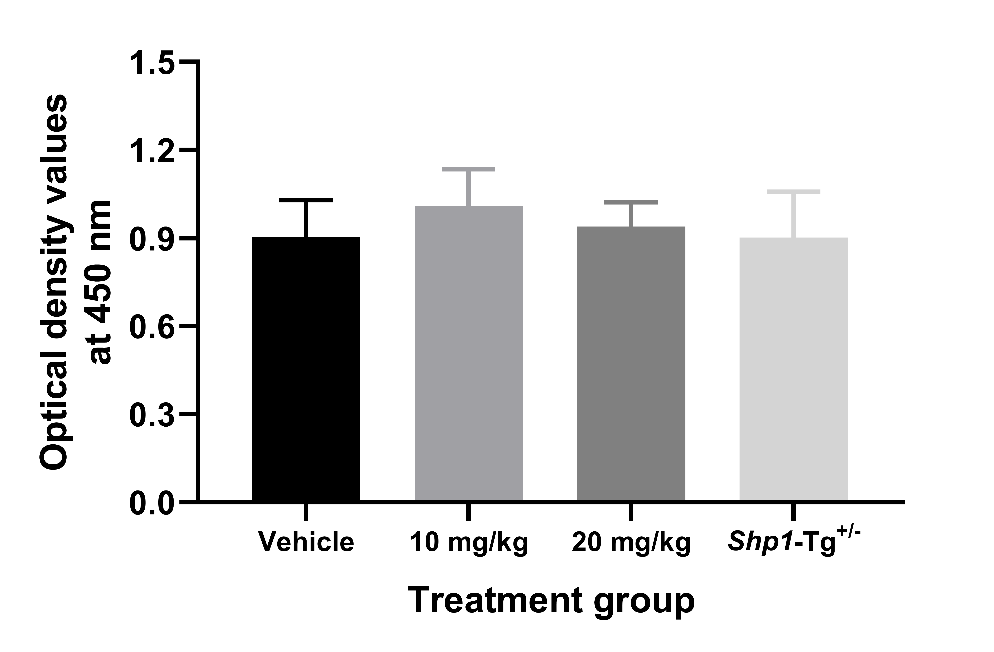


**Additional file 4. Serum anti-human PG antibody levels in PG-immunized and SHP-1 activator-treated or untreated mice.** Anti-human PG antibody content was measured in the sera of PG-immunized mice. WT mice underwent vehicle or regorafenib treatment, while Shp1-Tg^+/-^ mice did not receive treatment. Serum antibody titers are expressed as optical density (mean±SEM, n=5/group, one-way ANOVA).
